# Supplementary material for: Causal Relationship between Aging and Anorexia Nervosa: A White-Matter-Microstructure-Mediated Mendelian Randomization Analysis
Source: Biomedicines. 2024 Aug 16;12(8):1874. doi: 10.3390/biomedicines12081874 (PMC11351342; doi:10.3390/biomedicines12081874)
Supplement: Supplementary file 1 [file biomedicines-12-01874-s001.zip › Supplementary Figure/Figure S9/Leaveoneout plot of MR analysis for ubm-b-1950 to AN.pdf]

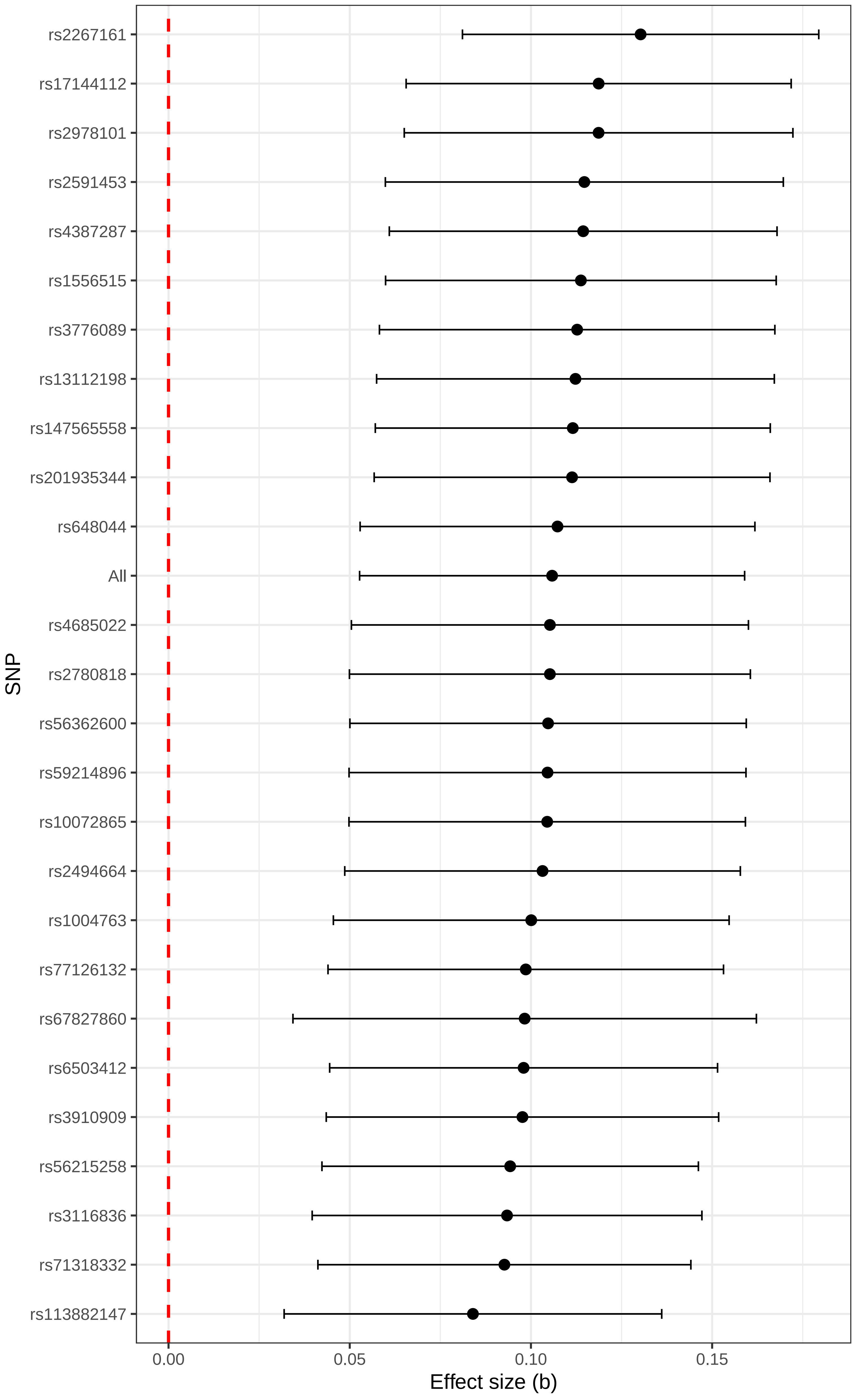
rs2267161
rs17144112
rs2978101
rs2591453
rs4387287
rs1556515
rs3776089
rs13112198
rs147565558
rs201935344
rs648044
All
rs4685022
rs2780818
rs56362600
rs59214896
rs10072865
rs2494664
rs1004763
rs77126132
rs67827860
rs6503412
rs3910909
rs56215258
rs3116836
rs71318332
rs113882147
SNP
0.00
0.05
0.10
0.15
Effect size (b)
